# Exploring linkages between research, policy and practice in the Netherlands: perspectives on sexual and reproductive health and rights knowledge flows

**DOI:** 10.1186/s12961-017-0201-0

**Published:** 2017-05-12

**Authors:** Billie de Haas, Anke van der Kwaak

**Affiliations:** 1 Independent researcher, Piri Reisplein 42, 1057 KH Amsterdam, the Netherlands; 2 Royal Tropical Institute, Mauritskade 63, 1092 AD Amsterdam, the Netherlands

**Keywords:** The Netherlands, Knowledge flows, Tacit knowledge, Knowledge platform, Sexual and reproductive health and rights

## Abstract

**Background:**

The attention to and demand for stronger linkages between research, policy and practice are increasing, especially in fields concerned with sensitive and challenging issues such as sexual and reproductive health and rights (SRHR). The study described in this article was conducted in the Netherlands among actors working in international development, especially the domain of SRHR. It explores the perceived flow of knowledge between research, policy and practice, the perceived impeding factors, and suggested strategies for improvement.

**Methods:**

A narrative literature review was performed and 28 key informants were interviewed between May and August 2015. Most interviewees were either active or passive members of Share-Net Netherlands, an SRHR knowledge platform. All interviews, which lasted 70 minutes on average, were recorded, transcribed verbatim and coded in MAXQDA.

**Results:**

Linkages between research, policy and practice are many and diffuse. The demands for and supplies of knowledge within and across the fields vary and do not always match, which is shown by participants’ research purposes and approaches. Participants identified various barriers to strengthening knowledge flows, including a lack of familiarity with practices in other fields, power relations and the undervaluation of tacit knowledge. They suggested a more visible and concrete demand for and supply of knowledge, the development of a joint knowledge agenda, more opportunities for the interdisciplinary creation of knowledge, and the development of a system for learning and sharing knowledge.

**Conclusion:**

This study shows the willingness to undertake, and the perceived advantages of, interdisciplinary dialogues and joint creation of knowledge to advance SRHR research, policies and practices. Whereas barriers to the flow of knowledge may maintain present understandings of knowledge and of whose knowledge is valid, enabling factors, such as interactions between research, policy and practice in knowledge-sharing activities, may challenge such perceptions and create an enabling environment for generating innovative knowledge and increasing knowledge use. Knowledge platforms are recommended to place more emphasis on sharing and documenting tacit knowledge through interdisciplinary dialogues, to address power relations and to set criteria for interdisciplinary funding.

## Background

The attention to and demand for stronger linkages between research, policy and practice are increasing, especially in fields concerned with sensitive and challenging issues such as sexual and reproductive health and rights (SRHR) [1]. Globally, academic institutes are being encouraged to conduct more societally relevant research, non-governmental organisations (NGOs) are required to show programme efficacy, and policies need to be transparent and evidence informed [2–8]. To achieve this, strengthened connections between actors in the different fields are needed, meaning that they share, use and contribute to each other’s knowledge development and products.

Often, linkages between research, policy and practice are considered one-directional, static knowledge flows, such as research uptake, whereby researchers disseminate their findings to inform policies and practices [9, 10]. However, it has been argued that dissemination of research findings alone is not sufficient to achieve social change and that it is important that various stakeholders share knowledge and jointly construct, or co-create, new knowledge [10, 11].

Actors within and across research, policy and practice understand and interpret knowledge differently. Knowledge is argued to be shaped and enacted in specific social contexts [12]. It is a subjective, contextual construction, rather than a mere object that can be easily transferred from one individual to another [13]. For instance, the construction of contraception as family planning may suggest that contraception should only be used by married couples for child spacing, and the word ‘condom’ may be associated only with the male condom rather than both the male and female condoms (e.g. (de Haas, B.: Sexuality Education in Uganda: Teachers’ Motivations, Reasoning and Vulnerability in an Abstinence-only Context, in preparation) and [14]).

Grey (Grey, 1996 in: Pathirage, Amaratunga, & Haigh, 2007, p.116) defines knowledge as “*the full utilisation of information and data, coupled with the potential of people skills, competencies, ideas, intuition, commitment and motivation*” [15]. This definition emphasises that knowledge includes both explicit knowledge and “*tacit understandings of what that information means and how it can be used*” [10] p.2. Explicit knowledge can be expressed in words and numbers, such as academic publications, which can be important to support policy and practice. For instance, Doug Kirby’s study published in 2007 showed that teaching of comprehensive sexuality education programmes does not lead to earlier sexual debut compared to abstinence-only programmes. His study was pivotal in lobbying and advocacy efforts for comprehensive sexuality education [16, 17]. Another example is the so-called ‘Dutch approach’ to SRHR, a progressive stand for SRHR in the Netherlands’ foreign policies supported by studies that show low rates of unintended pregnancies and abortions among young Dutch people due to easy access to contraception [18]. These examples suggest that knowledge that is generated and made explicit by academic researchers is considered the most legitimate type of knowledge to inform or support policy and practice. This indirectly implies that tacit knowledge, in general held more by policymakers and practitioners, receives less attention and, when made explicit, is considered less qualified or legitimate.

Tacit knowledge or understandings are more difficult to articulate than explicit knowledge [19]. Tacit knowledge is embedded in human beings based on experiences and can include perceptions, intuition and know-how [11, 15, 19, 20]. For instance, tacit knowledge may include practitioners’ know-how about the application of bottom-up approaches to enhance sexual agency when working with partners who are against homosexuality or feel that young people should not have access to contraception, or about training health workers to support women in post-abortion care in settings where abortion is illegal.

Explicit and tacit knowledge are complementary, and both are necessary in interactions between individuals to create new knowledge [19, 21]. Differences in jargon, discourse, publication formats and knowledge use are often considered barriers to enabling these interactions, leading to ‘gaps’ between the fields [22, 23]. For instance, whereas SRHR lobbyists and advocates may only be interested in the fact that comprehensive sexuality education does not lead to earlier sexual debut, researchers may be more interested in documenting theoretical foundations of such findings.

To increase the flow of knowledge – for instance, by creating and sharing knowledge – strong linkages between individuals operating in different domains are important. Such strong linkages are characterised by trust and long-term, close relationships. Repeated interactions focused on learning, such as through debates and dialogues, can strengthen these linkages and contribute to an understanding of each other’s contexts and knowledge needs [12]. This means that knowledge-sharing activities should have interactions between individuals and an understanding of each other’s contexts at their core.

The triangle of policy, research and practice is not always self-evident despite the willingness and engagement of different actors [23]. This article presents the findings of an exploratory study conducted in the Netherlands in 2015 among actors working in international development, especially the domain of SRHR. It aims to understand the type of activities that are needed to strengthen linkages between these actors and improve SRHR research, policy and practice. This will be done by studying the perceived flow of knowledge between policy, practice and research, the perceived impeding factors, and the suggested strategies for improvement.

A multitude of initiatives have been set up to improve knowledge use by strengthening flows of knowledge within and across the fields of research, policy and practice. Strengthening linkages through knowledge activities is known as, among other terms, knowledge management, knowledge translation, communities of practice and knowledge brokering [10, 24].

Share-Net International is one of the five knowledge platforms established by the Dutch Ministry of Foreign Affairs in 2013 to increase societally relevant research and research-informed policy and practice in the area of SRHR. Share-Net International sees itself as a community of practice, i.e. “*groups of individuals with shared interests that come together in person or virtually to tell stories, to share and discuss problems and opportunities, discuss best practices, and talk over lessons learned. Communities of Practice emphasize the social nature of learning within or across organizations*” [21]. The knowledge platform combines the expertise and strengths of Dutch organisations, southern partners and key international actors. Share-Net International consists of four country nodes, namely Share-Net Bangladesh, Share-Net Burundi, Share-Net Jordan and Share-Net Netherlands.

The establishment of Share-Net International in 2013 built on an existing SRHR network, called Share-Net Netherlands, which had been in place since 2001. Its long history brought along an existing infrastructure of secretarial support, returning activities and extensive commitment and dedication from members. Combined with the additional substantial funding received from the Dutch Ministry of Foreign Affairs in 2013, this created an enabling environment for the knowledge platform to flourish.

The present study focuses on Share-Net Netherlands, which has 47 members, including 24 NGOs, 7 universities, 3 other knowledge institutes, the Dutch Ministry of Foreign Affairs and 12 individual members [25]. To encourage learning across organisations, members of Share-Net Netherlands established 10 working groups; 7 of these are thematic working groups, the themes of which were chosen by members of Share-Net Netherlands. The themes were ‘Child marriage and teenage pregnancy’, ‘Comprehensive sexuality education’, ‘Contraception and abortion’, ‘Gender-based violence’, ‘SRHR and HIV integration’, ‘Sexual diversity’ and ‘Youth-friendly health services’. These themes show the emphasis of Dutch SRHR activities on youth, choice and voice. Discussions are regularly initiated about whether Share-Net Netherlands – for instance, its thematic working groups – should engage in lobbying and advocacy on behalf of its members. However, until now, it has always been concluded that Share-Net Netherlands should not be doing this, because its members do not all share the same views and opinions concerning sensitive SRHR issues such as abortion [26]. The three other working groups focus specifically on strengthening linkages between SRHR research, policy and practice – one on operational research, one on public–private partnerships and one on linking research, policy and practice.

Each working group comprises members working in research, policy and practice, and has developed its own knowledge strategy and organises related activities in which interdisciplinary dialogues and learning are core components such as annual thematic meetings [25]. Such interdisciplinary dialogues have already shown added value. For instance, students who presented their Master’s research at the annual ‘Linking students to policy and practice’ event have been invited by audience members to present their findings at the offices of UNFPA Brussels and the Dutch Ministry of Foreign Affairs. Another example concerns the ‘Small Grants’ studies that are funded by Share-Net International to stimulate small, practical and innovative research projects that complement lengthy, in-depth academic research. After a presentation of a Small Grants study about abuse during facility-based childbirth in Central Asia, a policymaker responded that they would address the issue on a visit to Central Asia the following week.

## Method

### Study design

**Fig. 1:**
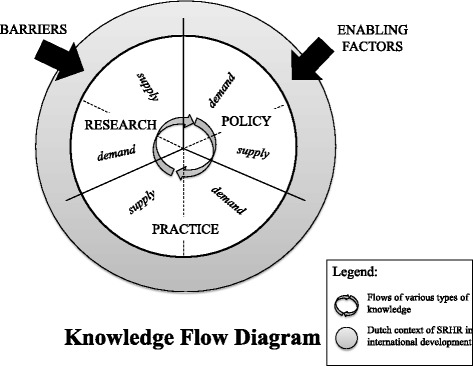
Conceptual model, based on Shaxson et al. [10]

The present study used key informant interviews to obtain people’s perceptions and a narrative literature review to embed those perceptions in the existing literature. The literature review was used to design the interview guide, develop the conceptual model and guide the interview coding and analysis. The conceptual model visualised the different knowledge flows between the fields of policy, research and practice, with each field assumed to have both a demand for and a supply of various types of knowledge. Within the study context different factors are assumed to facilitate or impede the flows of knowledge (Fig. 1).

The key informants were interviewed using a semi-structured interview guide, which was continuously tailored and fine-tuned during the data collection process [27]. The interview guide addressed the topics of the meaning of knowledge and evidence, knowledge use, perceptions on linking research, policy and practice in the context of the SRHR domain of the Dutch international development sector, needs and barriers regarding linking research, policy and practice, recommendations for strengthening linkages between research, policy and practice, and the envisaged role of Share-Net International as a knowledge platform.

Data were collected between May and August 2015. In total, 28 key informants participated in the study, most of whom were either active or passive Share-Net Netherlands members and working in senior positions at Dutch-based NGOs, co-funding organisations, the Ministry of Foreign Affairs, intermediary organisations, the public health service, or universities or other knowledge institutes (Table 1). As Table 1 shows, key informants could not be easily categorised according to a field of research, policy or practice, as researchers, policymakers, advocates and practitioners could be found working within the same organisations.

**Table 1:** Overview of key informants

Work environment	Number of key informants (male/female)	Job description
Academia and knowledge institutes	6 (1/5)	Post-doc researcher; Professor; Senior advisor; Senior researcher/lecturer (2); Assistant Professor
Policy	6 (2/4)	Ambassador; Policy advisor; Policymaker; Policy officer; Senior advisor; Trainee
Practice	9 (1/8)	Advocate; Coordinator; Manager (2); Policy advisor; Programme coordinator; Programme manager; Senior programme officer; Senior researcher
Intermediary organisation	3 (0/3)	Manager; Senior policy officer; Senior programme officer
Working in multiple fields	4 (1/3)	Consultant; Coordinator; Sexual and reproductive health and rights expert; Senior advisor/Professor
Total	28 (5/23)	

### Data analysis

All interviews, which lasted 70 minutes on average, were recorded and transcribed verbatim. They were then coded in MAXQDA, a qualitative data analysis program, using the following codes: (1) demand for and supply of knowledge; (2) barriers to the flow of knowledge; (3) present knowledge-sharing activities and other enabling factors; and (4) suggestions for improving the flow of knowledge. To allow comparison of the distinct fields, the data were analysed separately for the following fields or groups of people: (1) donors; (2) lobbying and advocacy; (3) policymaking/international NGOs; (4) programmes/implementation; and (5) research/education.

### Validation and ethical considerations

The key informants signed an informed consent form and afterwards received the transcript of their interview and a draft version of the report, on which they were requested to provide comments. To further validate the findings of the present study, and to encourage the participation of its members in the development of this report, Share-Net Netherlands organised a symposium entitled ‘Enter into dialogue: perspectives on linking research, policy and practice’. The preliminary findings were shared and discussed with the use of various interactive methods such as ‘Open Space Technology’. In addition, university students have further explored the feasibility of developing a joint knowledge agenda for the platform [28]. The Research Ethics Committee of the Dutch Royal Tropical Institute granted an ethics review waiver for the study (S 66), stating that “*the research consists of voluntary interviews with professionals that only cover information areas related to their duties and do not contain any personal questions. The participants can decide to decline or withdraw participation at any moment during the process without any consequences*” [29].

### Reflection on limitations and positionality

Most of the study participants were already aware of, or working at, the nexus of research, policy and practice. This may have created a more positive attitude towards linking the three fields.

Furthermore, the study may have been affected by the subjectivity and positionality of the researcher, or first author. At the time of data collection, the first author was a member of the Share-Net Netherlands Core Group and Chair of the Share-Net Netherlands working group ‘Linking research, policy and practice’. Based on her experiences as a Master’s and later as a PhD student, the first author has actively contributed to initiating activities focused on strengthening linkages between students and policy and practice. One of these activities is the annual ‘Linking students to policy and practice’ event, at which students present their research findings and speed date with practitioners and policymakers. At this event, students also receive an annually updated ‘Overview of SRHR knowledge questions from research, policy and practice’ posed by Share-Net Netherlands members that students could work on in their thesis [30, 31]. More recent activities include the NGO tour, whereby PhD students visit NGOs to discuss with practitioners how they could strengthen linkages between research and practice, and the development of a checklist that can be used to streamline communications in collaborations between students and NGOs, such as student internships.

As an active Share-Net Netherlands member, the first author’s emic or ‘insider’s perspective’ may have helped her go more deeply into the topic but may have also created some ‘blind spots’. Moreover, it may have prevented the key informants from sharing certain information, because they may have assumed that the researcher already knew about it or because they may have felt uncomfortable disclosing such information.

The second author has been an active member of Share-Net Netherlands from its start and chaired one of the working groups. She was also actively involved in the establishment of Share-Net International.

## Results

The following sections discuss the participants’ needs for knowledge, the factors they perceive as impeding knowledge flows, and their suggestions for improvement. It will become clear that although the literature suggests that knowledge is held by all stakeholders, the key informants did not always discuss knowledge in this way; most often they used words such as ‘research’ or ‘evidence’ and discussed one-directional flows of knowledge that make policy and practice more research-informed or research more relevant for policy and practice.

### Knowledge needs: demands for and supplies of various types of knowledge

#### Research purposes

There are various reasons for conducting research. For instance, key informants working at NGOs indicated that they demand knowledge, or research, to empower civil society, influence policies or build capacity. Depending on the purpose of the research, they will select a research method or approach:

“*I asked my partner: ‘Why do you need these data? What is your aim?’ He told me he wanted to push his partners to work more on governance and meaningful youth participation. So what would be the best way to approach this? Should we do quantitative research? Or an experiment?* […] *I notice that this makes me think differently about research. You try to figure out: what do partners need this research for?* […] *I start to explain research more in those ways than in scientific ways.*” (Senior researcher working in practice)

Depending on the research question, NGOs may perform research themselves, involve students or local researchers, or ask implementing organisations to conduct research themselves as a strategy for capacity building. Then, there are NGOs which involve senior academic researchers from leading academic institutes to increase the credibility of research findings. For instance, one practitioner felt that research needs to be conducted by leading academic institutes for policymakers to take it seriously:

“*You can do a quick and dirty evaluation, but if you need research findings for policy influencing, or for a difficult conversation, then you have to make sure that the research is conducted in a professional way.* […] *Some NGOs perform their own research, in which they let local NGOs collect their own data. I’m not convinced this will yield the best results.*” (Programme manager working in practice)

Practitioners and policymakers may ask academic researchers to conduct research that could inform their policy and practice. However, several researchers with experience of working in academia pointed out that the organisational incentive structure in academia encourages researchers to publish in peer-reviewed scientific journals only. Some key informants with experience of working in policy indicated that this supply of research output is not always relevant for policy and practice – for instance, when recommendations provided in peer-reviewed articles are too abstract to implement or when articles focus on explaining differences. In contrast, descriptive knowledge, such as programme evaluations or statistics describing the magnitude of SRHR issues, was perceived to be more relevant for influencing policies. A policy advisor explained the usefulness of empirical studies for policymaking:

“*To a policymaker, empirical knowledge is very important, and not so much the theoretical foundations of that knowledge, because you want to make policy based on a specific context, and that context matters, so that is what you want to know. That is a tension* [between research and policy]*, and I think that will never change.*” (Policy advisor working in policy)

At the same time, policymakers acknowledged that policymaking is not informed by evidence only, but also by other factors. For instance, they indicated that their policies are informed by international policies and agreements and that they are made within the frameworks and budgets set by politicians. They also indicated that, despite good access to academic research, time pressure can prevent policymakers from searching for academic research and, rather, make them rely on information that is readily available, such as feedback from practitioners that implement their policies or other information received from actors in their network. A policymaker indicated that, often, Dutch research on SRHR does not influence Dutch policymaking on SRHR directly but via international technical agencies that set international guidelines on SRHR:

“*We need to realise that the Netherlands is a small country. It would be unrealistic to think that Dutch research influences Dutch policies. That’s not how it works. We have a worldwide policy development informed by evidence which is translated into international guidelines and theories. As the Netherlands, we need to take these into account and try to see where we can add value – how we can contribute.*” (Senior advisor working in policy)

Similarly, the same policymaker mentioned that Dutch SRHR policy focuses on young people because this niche is not covered by many other countries and enables the Netherlands to make a difference with the limited budget available.

To meet the knowledge needs of policy and practice, several researchers indicated that, despite incentives for publishing in scientific journals, they do disseminate their findings in various formats intended for various audiences, including factsheets or policy briefs to reach policymakers. However, one researcher felt that there is a potential pitfall of focusing knowledge dissemination too much on reaching policymakers through policy briefs only:

“*If we start writing small policy briefs only* […] *I don’t like it. Policy briefs, is that all we’re striving for? And I think, I hope, there are certain policymakers who are willing to read an academic article* […]*. The products resulting from research and knowledge generation should be diverse, and it should not just be one standard product with one format. I don’t believe in it.*” (Senior advisor working in research)

#### Research approaches

Several key informants working in research and practice felt that Dutch NGOs are now being held more accountable for how they spend their funding. Although most of them saw this as a positive development, some questioned the way this has been translated into the monitoring and evaluation conditions set by funding agencies. For instance, donors may ask NGOs to perform big impact evaluations and to collect extensive quantitative data on a broad spectrum of indicators. Various key informants argued that other research approaches, such as process evaluations using qualitative research methods, could be more useful and appropriate as they allow tacit knowledge, such as best practices and lessons learned, to be documented. For instance, the following practitioner preferred to see less, but more in-depth, data collection, including time to learn from and reflect on such lessons learned and to adapt programmes accordingly:

“*There is this bureaucratic system of having many results and aggregating results to a level where it loses its meaning.* […] *This focus on numbers* […] *doesn’t tell you anything about the quality of what has been achieved.* […] *Many small studies are performed in too short a period of time by researchers who have little background in research.* […] *Instead, one big research project with a well-developed research question would have helped us a lot more.*” (Programme coordinator working in practice)

Additionally, some researchers have encountered donors demanding their own preferred methods and approaches and lacking faith in innovative research methods suggested by researchers:

“*I find often there is a lot of sort of lip service to things like children’s voices, but then there is no actual support for doing that, you know, for doing that in your research, or making that matter in your policies. It tends to be very tokenistic in the end.*” (Senior researcher working in research)

In such cases, researchers felt that their skills and tacit understandings of how to conduct quality research using a variety of methods and approaches could have met the knowledge requirements of policymakers and practitioners, but that certain barriers, such as disputes about research approaches, prevented them from collaborating.

### Barriers to the flows of knowledge

The key informants in this study mentioned various barriers in the current environment for knowledge flows between research, policy and practice.

#### Discrepancies in duration and cycles

Some key informants considered that academic research, which often takes a few years to generate, might not be appropriate for answering knowledge questions from policy and practice, which, they say, often need to be answered in the short term. For instance, the following policy advisor preferred short-term research:

“[Academic studies] *are often long-term trajectories. What may be more interesting are studies conducted by Master’s students: they… conduct their research in three months, and then the result is available. At least at the Embassy where I worked, we used to make use of such studies a lot because it allowed us to steer the type of research, to facilitate the process, and then the results were available much faster.*” (Policy advisor working in policy)

However, other participants questioned whether this is true and argued that a distinction should be made between policy questions, which could be long term, and political questions, which could be short term. For instance, one policymaker said that it is actually important for SRHR policy to be consistent in the long term to achieve positive change:

“*Because the Dutch SRHR policy has been consistent for the last 10 years, the international world also knows that these are the Dutch priorities* […] *And I think it’s good; our SRHR agenda is one that needs a long term.* […] *It often takes a generation before social and cultural norms start changing, like accepting that access to contraception or sexuality education for young people is normal* […]*. Our SRHR agenda is full of such social norms, plus all the opposition involved.*” (Senior advisory working in policy)

Researchers and practitioners also emphasised that it takes time to achieve cultural and social change on SRHR issues. Some indicated that fixed project cycles of 4 years are often too short to implement programmes effectively, especially when this includes setting up a process for various stakeholders to work together. Both researchers and practitioners felt that, if project cycles lasted longer, more sustainable change could be achieved, and it would become easier to measure these changes.

#### Incentive structures

In addition to a lack of incentives for academics to disseminate research findings in other ways than peer-reviewed articles, a lack of incentives was also mentioned as a barrier to learning and sharing knowledge with other professionals within and across the fields of research, policy and practice. For instance, this practitioner explained that incentive structures in NGOs encourage them to prioritise writing proposals:

“*People have other things on their minds right now* […] *if they need to choose between writing a project proposal or learning…* […] *Yes, they will tell you that learning is a priority, but I would like to see what that means in terms of time being invested.*” (Programme manager working in practice)

#### Lack of familiarity and expectations

One former policymaker felt that a barrier to evidence-informed policymaking is the diverse research field for SRHR in the Netherlands, whereby different universities and research groups are engaged in aspects of SRHR, which makes it difficult for outsiders to know where to obtain specific information. A practitioner also mentioned that she would like to collaborate more with Dutch universities but does not know exactly which university is doing what and where there could be opportunities for collaboration.

A related barrier is prejudices and expectations about other professionals and a polarisation of their different approaches. For instance, a key informant working in policy felt that researchers only care about publishing, and a researcher considered that many practitioners only perceive research to be valid if it takes a positivistic approach:

“*Sometimes those who are working in practice or in policy,* […] *they want certain kinds of information, numbers that reinforce that* [programme efficacy]*, right? And yet there are some instances in which the kinds of programmes that they want to do don’t lend themselves well to this sort of quantitative or very positivist kinds of research. So you have to sort of get past those kinds of barriers.* […] *In fact, sometimes I think* [they] *question the quality of something that isn’t positivist, right?*” (Senior researcher working in research)

Yet, many of the interviewees work at the nexus of these fields, which suggests that their approaches may not always differ that much in reality:

“*There are so many people doing the same things, only placing different accents. Sometimes the barriers or distances seem big, but in reality they are not.*” (Senior advisor working in research)

#### Power relations

Several key informants mentioned the role of power relations. For instance, one key informant felt that it would be good to acknowledge the role of power in flows of knowledge:

“*I think part of the problem is not realising how political it is. It’s so… these areas are so political and so much about power and so much about who gets what in terms of wealth and attention and rights. If you don’t have that rooted in your thinking, it’s not going to change, I don’t think.*” (Consultant working in policy and practice)

Some Share-Net Netherlands members indicated that power relations play a role within the knowledge platform. For instance, their membership and participation may be motivated by their political or strategic agenda in relation to the Dutch government and other SRHR organisations. Furthermore, some members perceived competition and competing interests between members:

“*We are all enormously competitive because we are trying to get funding from the same donors.* […] *I find it difficult because the knowledge platform is for sharing and learning. You have common goals, but I notice the competition is constraining.* […] *You notice it, for instance, in the agenda setting because each member wants to attract attention to their own niches.*” (Programme manager working in practice)

Another power-related barrier identified by key informants is a donor-driven agenda for research and practice, which they felt does not always prioritise needs based on evidence and works against the long-term implementation necessary for programmes to have impact. Rather than focusing on programmes that have proven their effectiveness, they may feel urged to focus on innovative projects:

“*I think our old way of working is still very good: working on many components, involving everyone: an evidence-based approach.* […] *But you see us moving away from it, because there is less funding for this way of working. So you’re donor-driven; you start developing new approaches.* […] *This shows you that the donor decides. And for the Dutch practitioners and researchers working on SRHR, this means the Ministry plays a very big role.*” (Senior programme officer working in practice)

### Suggestions for improving the flow of knowledge

Reducing the barriers discussed by the key informants can contribute to an enabling environment for linking research, policy and practice. This section discusses some suggestions provided by the key informants to increase the flow of knowledge between research, policy and practice, to make more progress and improve the sustainability and impact of their activities.

#### A visible and concrete demand for and supply of knowledge

The participants indicated that they are curious about the knowledge demands and supplies of other professionals working on SRHR:

“*Asking policymakers:* […] *‘What kind of knowledge do you want out of the knowledge platforms? To what extent do you have a need to use knowledge for policymaking?’* […] *to make this more explicit.*” (Senior programme officer working in practice)

To share their demand with research and practice, a policymaker stated that, first of all, this means that policymakers should make their own demand for knowledge more visible and concrete. The participants would also like to know how policymakers, researchers and practitioners use knowledge, which cycles they go through and how other professionals can connect to the various stages of such cycles to strengthen linkages between the fields. For instance, a researcher observed that the Dutch government has not yet made PrEP available in the Netherlands despite research that shows it is effective in preventing HIV infection in high-risk groups. She would like to understand the different factors that are being considered when developing policy and practice and how research plays a role in that process:

“*They* [the Dutch Ministry of Foreign Affairs] *chose specific countries. Why did they choose those countries?* […] *The same goes for NGOs: I would like to know why they focus on certain programmes. Do they even know whether they are effective or not?*” (Post-doc researcher working in research)

#### Develop and push a joint knowledge agenda

The key informants felt that the development of a joint knowledge agenda, which maps the demand for and supply of knowledge in the various fields, will enable stakeholders to see where they can complement or collaborate with others and how value can be added to the knowledge that is already there. A researcher working in practice said:

“*Which research agenda is relevant? After having established this, you can let people go off to do their own thing, compete with one another, but do have this conversation: ‘Where do we as Dutch institutes add value? What do we want to push? Which research agenda do we want to follow or push with donors?’*” (Senior advisor working in practice)

A joint knowledge agenda can help discuss how Dutch SRHR knowledge can add value and proactively link to the international knowledge base. For instance, a policymaker from the Dutch Ministry of Foreign Affairs explained how she had stimulated linking Dutch research to the international knowledge base by arranging for a Dutch professor to present the findings of their research programme on HIV and AIDS to the World Health Organization in Geneva. A joint knowledge agenda may also counteract feelings of dissatisfaction among practitioners and researchers about work being donor-driven, by being more proactive rather than reactive to donor policies.

#### Increase opportunities for strengthening linkages between research, policy and practice

Interviewees provided some recommendations for overcoming discrepancies between research, policy and practice with regard to differences in project cycles and project durations. First, it was argued that a variety of short- and long-term research studies can be complementary and can help answer the variety of knowledge questions – for instance, by asking students to answer short-term knowledge questions. Second, it was suggested that there should be more opportunities and time allocated in project proposals for learning, reflection and adaptation during programme implementation itself, including research projects, where findings can be shared and discussed during the project, rather than only at the end. Third, there could be greater efforts to link research to policy and practice after research findings have been published:

“*Usually it is only after research financing stops that research uptake starts. It can be years later that scientific research starts to manifest itself somewhere and is taken up by accident. And that is a shame.* […] *I would ask to run these projects for a longer period of time, so researchers can stay active and continue developing based on what they have collected.*” (Manager working at an intermediary institution)

Another recommendation provided was to link knowledge more consciously to the stages of policy and project cycles when knowledge input is required, and to make research, policy and practice cycles more compatible – for example, publishing policy briefs ahead of international conferences.

Interviewees felt that there is a need to create more mutual understanding between professionals working in research, policy and practice and to establish incentives for working together, such as integral funding and donors setting criteria for collaborations. A key informant working at an intermediary organisation thought that research processes involving interdisciplinary stakeholders can have more impact than the findings emerging from the project:

“*I think a research process involving various stakeholders* […] *putting energy in, making these parties collaborate well and the expertise being developed during such a process can be useful for the broader environment. I think such a process can have more impact than the findings coming out of the project. It’s the way of working, the way of thinking and the interaction between stakeholders that can be enriching for research, policymakers and practitioners.*” (Manager working at an intermediary institution)

Other suggestions involved interdisciplinary working. For instance, a researcher felt that policy and practice can become more evidence-informed if researchers start working in these fields after having finished their PhD. A policymaker and a researcher suggested that one-day or more long-term internships, where professionals are introduced to the daily work of professionals in other fields, may help to overcome the lack of familiarity and develop an understanding of each other’s knowledge demands. Policy and practice could also become more integrated in the educational curriculum of universities.

#### Develop a system for learning and sharing knowledge to increase knowledge use

It was suggested to have a greater focus on process learning, to discuss how implementation processes can be improved.

Several participants indicated that their organisations are looking for promising practices of linking research and practice – for instance, sharing experiences about conducting operational research and how this can support capacity building, but also exchanging best practices of involving students in organisations such as through internships.

A researcher suggested the development of a system for learning and sharing knowledge based on a variety of knowledge-sharing activities that will lead to joint creation and innovation:

“*So there should be a system for giving direction to each activity, to make it useful and carry it along.* […] *There should be more temporality in these things: one after the other, it needs more time, not doing everything at once* […] *to understand how these various strategies influence each other and should connect.*” (Senior advisor working in research and practice)

Another participant with experience in working in research, policy and practice thought that this requires setting up a process which includes participation, interaction and space for reflection and discussion:

“*You have to set up processes to make it happen.* […] *The process of linking, and not even new knowledge. I mean, expecting new knowledge is a bit much in that short time, but actually getting… getting those processes working better, I think that’s what it really should be about.* […] *That doesn’t just happen by being, putting people in a room; you’ve got to really pursue that, I think. I don’t mean it in terms of money; I mean it in terms of feeling that they are part of a more effective community.*” (Consultant working in policy and practice)

To create such a system, it was suggested, for instance, that researchers and practitioners could be encouraged to formulate recommendations and a plan of action together in a participatory process, to regard research uptake as part of the research process, and to have discussions about documents being published. Several study participants felt the need for a knowledge broker to ensure that such linkages are made.

## Discussion

The Dutch field of actors working on SRHR in international development appears relatively small in terms of their number and their geographic proximity, which allows for relatively easy face-to-face interactions, including with policymakers at the Dutch Ministry of Foreign Affairs. The field also appears small in terms of its focus on sensitive themes, including young people, abortion and sexual diversity within the broad topic of SRHR. Although members may differ in their views and opinions, as a knowledge platform, Share-Net Netherlands seems to be characterised by the active participation of members who share a common commitment and dedication to the topic of SRHR, whether addressed from research, policy or practice. This situation encourages collaborations and relationships, which are found to facilitate linkages between research, policy and practice [32].

However, also in this enabling environment, key informants could experience a mismatch between the supply of and demand for knowledge between the fields of research, policy and practice due to divergent project cycles, research methods and outputs. These findings confirm the barriers found in other studies about evidence-informed policymaking [32]. A lack of familiarity with and expectations about other professionals’ supply of and demand for knowledge strengthened these perceived barriers between the fields. At the same time, most study participants appeared to be working at the nexus of research, policy and practice, which indicates that the supply of and demand for knowledge between the fields of research, policy and practice do find each other and may actually overlap.

To optimise knowledge flows within the Dutch field of SRHR in international development, the study participants made various suggestions for improving the flow of knowledge. First, participants suggested making the demand for and supply of knowledge more visible and concrete. Whereas systematic literature studies and an open-access knowledge base can contribute to a more focused and visible supply of explicit knowledge, interdisciplinary interactions can contribute to a more focused supply of tacit knowledge. The literature shows that joint creation of knowledge is a prerequisite to achieve innovation and that tacit knowledge is the most important source for innovation, which can only have impact if it is made explicit and shared with others in interdisciplinary interactions [10, 11]. As a Community of Practice, Share-Net International recognises and acts on the importance of tacit knowledge by organising interdisciplinary interactions for sharing knowledge [21]. The findings suggest that there is interest in scaling up activities focused on interdisciplinary interactions and making tacit knowledge more explicit.

Second, the development of a joint knowledge agenda was suggested. If Share-Net Netherlands is to initiate the development of such an agenda, a first step would be to start a dialogue between Share-Net Netherlands members to discuss prejudices, expectations and power relations between the fields of research, policy and practice, and to make the differences in the demand for and supply of knowledge more visible [28]. This will require a safe environment in which stakeholders feel free to share their perspectives. Following a process of evidence-based priority setting, taking ethical considerations into account, for example, providing special attention to underrepresented groups, may contribute to creating such a safe environment [33–35].

Third, taking into account the barriers underlying the specific context of the Dutch field of SRHR in international development could encourage opportunities for strengthening linkages between research, policy and practice. Although participants discussed how changing incentive structures could encourage researchers to conduct more policy- and practice-relevant research and could encourage researchers and practitioners to engage in knowledge-sharing activities, it remained unclear which incentives policymakers need to become more actively involved in knowledge-sharing activities, and how knowledge should be translated and shared for policymakers to use it. Research suggests that the involvement of policymakers in the research process of formulating proposals and developing recommendations could encourage them to use the findings [36]. On the other hand, it should also be realised that research finds policy via practice, such as in the case of Dutch NGOs lobbying for PrEP availability [37].

A fourth suggestion was to develop a system for learning and sharing knowledge within Share-Net Netherlands. The knowledge platform can initiate knowledge-sharing activities and intensify activities in accordance with the ‘knowledge spiral’. This can help to evolve knowledge by sharing tacit knowledge and making it more explicit. Opportunities can be found in intensifying activities focused on (1) originating, for example, sharing tacit knowledge between individuals, such as through short-term interdisciplinary internships; (2) conversing, for example, bringing small interdisciplinary working groups together to exchange tacit knowledge through dialogues; (3) documenting, for example, documenting tacit knowledge about lessons learned and best practices and how to adapt them to local contexts, indeed, a learning agenda may help prioritise tacit knowledge discussions; and (4) internalising, for example, by discussing such findings in workshops. This ‘knowledge spiral’ is in accordance with key informants’ suggestions to strenghten and expand a system for learning and sharing knowledge that does not stop at documenting knowledge but continues to evolve this knowledge in an upward spiral [11]. This means not only working together in participatory ways but also acknowledging temporality and initiating consecutive activities such as including the development of a plan of action in each research project. The three Share-Net Netherlands working groups that focus specifically on strengthening linkages between SRHR research, policy and practice can be meaningful in optimising such knowledge systems.

Finally, the suggestion to increase attention to implementation processes by sharing promising practices and lessons learned between organisations and documenting them will help to ‘globalise local knowledge’. Share-Net International’s future activities could focus on how linkages between the knowledge platforms in the four country nodes might be strengthened.

### Implications for practice

Knowledge platforms can play a brokering role between research, policy and practice by improving the knowledge flows between these fields. A variety of knowledge activities can help to match the demand for and supply of knowledge between actors within and across the fields, especially activities focused on interdisciplinary dialogues to share and jointly create knowledge and to document knowledge in various formats intended for various audiences, including factsheets or policy briefs, to improve knowledge use in decision-making. More emphasis could be placed on sharing tacit knowledge and developing a system for evolving knowledge by developing a plan of action for each activity [11]. Furthermore, knowledge platforms and funding agencies are recommended to contribute to an enabling environment for knowledge flows by addressing barriers, such as power relations, and supporting enabling factors by creating opportunities for interdisciplinary funding. Organisations are recommended to create incentive structures for their employees, including the time, resources and power to engage in knowledge-sharing activities, reflect on lessons learned and adjust programmes accordingly.

## Conclusions

This article explored how, in the Netherlands, actors working in international development, especially the domain of SRHR, perceive the flow of knowledge between research, policy and practice, impeding factors, and strategies for improvement.

The findings suggest that some types of knowledge, such as ‘evidence’ resulting from academic research, may be considered more legitimate than other types of knowledge, and that it is not always easy to be aware of the knowledge held by practitioners and policymakers. The study participants felt that it is important to recognise that, like knowledge held by practitioners and policymakers, academic research is also both valuable and bound by limitations such as, for example, bound by what can and cannot be researched. Furthermore, they also felt that tacit knowledge is currently undervalued and represents an opportunity for creating impact and innovation.

This study has shown the willingness to undertake, and the perceived advantages of, interdisciplinary dialogues and joint creation of knowledge to advance SRHR policies and practices. Whereas barriers to the flow of knowledge, such as power relations and a lack of familiarity, may maintain present understandings of knowledge and of whose knowledge is valid, enabling factors, such as interactions between research, policy and practice in knowledge-sharing activities, may challenge such perceptions and create an enabling environment for generating innovative knowledge and increasing knowledge use.

## Abbreviations

NGO: non-governmental organisation
SRHR: sexual and reproductive health and rights.
